# Assessment of wavelength-dependent parameters of photosynthetic electron transport with a new type of multi-color PAM chlorophyll fluorometer

**DOI:** 10.1007/s11120-012-9758-1

**Published:** 2012-06-23

**Authors:** Ulrich Schreiber, Christof Klughammer, Jörg Kolbowski

**Affiliations:** Julius-von-Sachs Institut für Biowissenschaften, Universität Würzburg, Julius-von-Sachs Platz 2, 97082 Würzburg, Germany

**Keywords:** *Chlorella*, ETR, Functional absorption cross section of PS II, *O*–*I*_1_ fluorescence rise, PAR, Photoinhibition, Synechocystis

## Abstract

Technical features of a novel multi-color pulse amplitude modulation (PAM) chlorophyll fluorometer as well as the applied methodology and some typical examples of its practical application with suspensions of *Chlorella vulgaris* and *Synechocystis* PCC 6803 are presented. The multi-color PAM provides six colors of pulse-modulated measuring light (peak-wavelengths at 400, 440, 480, 540, 590, and 625 nm) and six colors of actinic light (AL), peaking at 440, 480, 540, 590, 625 and 420–640 nm (white). The AL can be used for continuous illumination, maximal intensity single-turnover pulses, high intensity multiple-turnover pulses, and saturation pulses. In addition, far-red light (peaking at 725 nm) is provided for preferential excitation of PS I. Analysis of the fast fluorescence rise kinetics in saturating light allows determination of the wavelength- and sample-specific functional absorption cross section of PS II, Sigma(II)_λ_, with which the PS II turnover rate at a given incident photosynthetically active radiation (PAR) can be calculated. Sigma(II)_λ_ is defined for a quasi-dark reference state, thus differing from σ_PSII_ used in limnology and oceanography. Vastly different light response curves for *Chlorella* are obtained with light of different colors, when the usual PAR-scale is used. Based on Sigma(II)_λ_ the PAR, in units of μmol quanta/(m^2^ s), can be converted into PAR(II) (in units of PS II effective quanta/s) and a fluorescence-based electron transport rate ETR(II) = PAR(II) · Y(II)/Y(II)_max_ can be defined. ETR(II) in contrast to rel.ETR qualifies for quantifying the *absolute* rate of electron transport in optically thin suspensions of unicellular algae and cyanobacteria. Plots of ETR(II) versus PAR(II) for *Chlorella* are almost identical using either 440 or 625 nm light. Photoinhibition data are presented suggesting that a lower value of ETR(II)_max_ with 440 nm possibly reflects photodamage via absorption by the Mn-cluster of the oxygen-evolving complex.

## Introduction and instrument methodology

Since its introduction, now more than 25 years ago (Schreiber et al. 1986), pulse amplitude modulation (PAM) fluorometry in conjunction with the saturation pulse (SP) method has become a routine tool for non-invasive assessment of photosynthetic electron transport in higher plants, algae, and cyanobacteria (Schreiber 2004; reviews in Papageorgiou and Govindjee 2004). In particular, PAM-measurements of maximal and effective PS II quantum yields via the fluorescence parameters *F*_v_/*F*_m_ = (*F*_m_ − *F*_o_)/*F*_m_ and Y(II) = ($F^{\prime}_{\text{m}}$ − *F*)/$F^{\prime}_{\text{m}}$ (Genty et al. 1989) have proven of considerable practical relevance. In most applications, *relative* changes of these parameters are of primary interest, e.g., caused by photoinhibition or other types of environmental stress. The same is true for the ETR parameter, derived from Y(II), which provides a relative measure of linear electron transport rate (Schreiber et al. 1994). Determination of *absolute* values of *F*_v_/*F*_m_, Y(II) and ETR is complicated by non-PS II fluorescence (e.g., originating in PS I or in the phycobilisomes) and by the difficulty to determine the quantum flux density (or photon fluence rate) of *PS II*-*absorbed* actinic light (AL), which depends on chlorophyll content and the PS II absorption spectrum as well as on the color of the applied light. Information on PS II absorption is a prerequisite for assessment of photosynthetic electron transport rates in optically thin suspensions of algae and cyanobacteria via chlorophyll fluorescence measurements, which is particularly true for estimation of primary productivity by phytoplankton in natural waters. Hence, it is not surprising that the methodology for determination of *PS II-specific light* *absorption* and assessment of *absolute ETR* values has been particularly advanced by researchers in oceanography and limnology (Falkowski and Raven 2007; Kolber et al. 1998). In the study of leaves, which absorb almost all incident photosynthetically active radiation (PAR) most researchers simply have been assuming that 84 % of incident PAR is absorbed (Björkman and Demmig 1987), being evenly distributed between PS I and PS II. This approach has been justified by satisfactory agreement with simultaneous measurements of the rate of CO_2_ fixation (Genty et al. 1989; Krall and Edwards 1990; Siebke et al. 1997).

While determination of PS II absorption in leaves is complicated by wavelength-dependent intra-leaf light gradients (Vogelmann 1993), it can be realized in a straight forward way in optically thin suspensions via chlorophyll fluorescence measurements. Ley and Mauzerall (1982) introduced the term of the functional absorption cross section of PS II, σ_PSII_, which is measured via the flash-intensity saturation curve of the fluorescence increase induced by single-turnover (ST) flashes. This approach has been applied extensively and further developed by Falkowski and co-workers (Falkowski and Kolber 1995; Falkowski et al. 2004; Falkowski and Raven 2007; Kolber et al. 1998). The development from the original pump-and-probe method toward fast repetition rate (FRR) fluorometry has been converging with parallel developments in PAM fluorometry (Jakob et al. 2005; Kolbowski and Schreiber 1995; Neubauer and Schreiber 1987; Schreiber 1986; Schreiber et al. 1993, 1995, 2011). With current instrumentation, both approaches allow measurements of the fluorescence rise induced by strong AL, estimation of the functional absorption cross section of PS II and assessment of maximal and effective PS II quantum yields after single- or multiple-turnover (MT) closure of the PS II acceptor side.

In contrast to leaves, which show relatively flat absorption spectra, dilute suspensions of unicellular algae and cyanobacteria display pronounced wavelength-dependent differences of PS II absorption, which are reflected in characteristic fluorescence excitation spectra, representing the “finger-prints” of the various types of PS II antenna pigment-systems (cyanobacteria, cryptophytes, green algae, diatoms/dinoflagellates). Multi-wavelength PAM fluorometers have been developed to estimate the content of various pigment-groups of phytoplankton in mixed natural waters (Beutler et al. 2002; Kolbowski and Schreiber 1995), by deconvolution of the overall signal into several components, based on “reference spectra” for the major pigment-groups. However, as was pointed out by Jakob et al. (2005), reliability and accuracy of this approach are limited by potential differences between σ_PSII_ of the various types of phytoplankton in natural waters and the laboratory-grown cultures used for measurements of “reference spectra”. Furthermore, not only the differences in σ_PSII_ between the various types and adaptation states of phytoplankton have to be considered but also the wavelength dependence of σ_PSII_.

While the theory of FRR fluorometry (Kolber et al. 1998) in principle does account for species and wavelength dependence of σ_PSII_, in practice, in situ measurements normally are carried out with naturally occurring mixed samples and a single color of measuring and AL, so that the obtained parameters *F*_v_/*F*_m_ and σ_PSII_ cannot give specific information. Hence, relative changes in these parameters can be interpreted only if changes in relative contents of different pigment types can be excluded. In most FRR studies, blue light has been used, as this approximates the spectral light quality in marine environments, the PS II absorption of which differs considerably between different types of phytoplankton. This aspect is dealt with in a recent report on FRR measurements by Suggett et al. (2009) who state: “It is now becoming clearer that in situ values of Fv/Fm and σ_PSII_ also contain taxonomic information” and “The magnitudes of variability in Fv/Fm and σ_PSII_ driven by changes in phytoplankton community structure often exceed that induced by nutrient limitation.”

Most PAM fluorometers just provide one color of pulse-modulated measuring light (ML) (normally red or blue), with the option of applying AL of any spectral composition, including natural sun light. With the XE-PAM (Schreiber et al. 1993), which employs xenon-discharge flashes for both ML and saturating ST flashes, the colors of measuring and AL can be defined with the help of optical filters. While this instrument allows estimation of σ_PSII_ by the pump-and-probe method, this approach has not been much used, as it is time-consuming and requiring considerable background knowledge and experimental skill. The phyto-PAM (Jakob et al. 2005; Kolbowski and Schreiber 1995) employs four different colors for ML, but just one color of AL (red) and, hence, does not allow estimating the wavelength-dependent σ_PSII_. The microfiber-PAM (Schreiber et al. 1996) offers four different colors for measuring and AL. This device, however, lacks the time resolution for assessment of rapid rise kinetics, required to estimate σ_PSII_. The same is also true for a recently introduced multi-color PAM fluorescence imaging system (Trampe et al. 2011). Finally, the very recently developed multi-color-PAM (Schreiber et al. 2011) provides six different colors of ML and six different colors of AL, all of which qualify for highly accurate measurements of fast induction kinetics and assessment of wavelength-dependent *F*_v_/*F*_m_ and functional absorption cross section of PS II. This new device is the topic of the present communication.

In practice, for correct determination of the wavelength-dependent σ_PSII_ via the fluorescence rise kinetics in strong light, a number of physiological factors have to be considered, which affect the rise kinetics and, hence, had to be accounted for in the development of the methodology: (1) state of light acclimation of the sample (state 1 or state 2); (2) redox state of the PS II acceptor side, including the PQ-pool; (3) limitation of PS II turnover at very high light intensities (fluorescence rise within about 100 μs) by a non-photochemical loss process (Rappaport et al. 2007); (4) quenching of fluorescence at the so-called *I*_1_-level (Samson et al. 1999; Schreiber 1986, 2004; Schreiber and Krieger 1996). Consideration of these factors has led to a somewhat modified approach for determination of the functional absorption cross section of PS II, with respect to the pump-and-probe and FRR methods. The measurement is carried out with the sample being in a defined quasi-dark (+far-red, FR)-adapted “reference state” using relatively moderate actinic intensities (fluorescence rise within about 1 ms), with maximal fluorescence yield (i.e., *I*_1_-level at saturation of photochemical phase) being induced at the end of the rise curve by a saturating ST flash. Therefore, the functional PS II absorption cross section measured with the multi-color-PAM is valid only for the reference state in which it was measured and any changes of PS II efficiency occurring, e.g., during illumination are assumed to be covered by corresponding changes in the effective PS II quantum yield, Y(II). For this reason, to avoid confusion with the previously defined σ_PSII_, which changes during illumination and in response to chlororespiratory electron flow (Koblizek et al. 2001), the wavelength-dependent functional PS II absorption cross section determined with the multi-color-PAM is called Sigma(II)_λ_.

For correct assessment of Sigma(II)_λ_, it is essential that the quantum flux density of the incident PAR is homogeneous, which can be realized only at rather low chlorophyll content (below about 500 μg Chl/L in suspensions), thus excluding straight forward measurements with leaves. However, even with optically dense objects valuable information can be obtained by application of different colors of light, differing in depths of penetration, a topic that recently has received considerable attention (Oguchi et al. 2011; Rappaport et al. 2007; Takahashi et al. 2010; Terashima et al. 2009), with the first and the two latter studies concentrating on the wavelength dependence of photoinhibition.

There has been general agreement that PS II is the primary target of photoinhibition and can be measured via the decrease in *F*_v_/*F*_m_ (Demmig-Adams and Adams 1992). The molecular mechanism of the primary photodamaging reaction, however, is still controversial. Recently, the so-called two-step hypothesis has been advanced (Hakala et al. 2005; Nishiyama et al. 2006; Ohnishi et al. 2005), which suggests that the primary step involves damage of the oxygen-evolving complex (OEC), when the Mn-cluster dissociates after absorption of UV or blue light; donor-side limitation of PS II is supposed to induce secondary damage upon light absorption of PS II pigments, due to the increased life time of P680^+^ and the resulting formation of singlet oxygen. This hypothesis is supported by action spectra of photodamage to PS II with peaks in the UV-A and blue region, resembling those of model manganese compounds and differing considerably from PS II absorption spectra (Hakala et al. 2005).

Whereas measurements of the wavelength dependence of photoinhibition in leaves are complicated by intra-leaf light gradients and fluorescence reabsorption, it can be investigated in a straight forward way in optically thin suspensions. As this topic is close to the heart of Osmond (1981, 1994) to whom this contribution is dedicated, in addition to the technical and methodological aspects of the multi-color-PAM also an application of this new device in the study of the wavelength dependence of photoinhibition will be presented. In this application, use of the possibility is made to adjust defined rates of quanta absorption by PS II with blue and red lights in a dilute suspension of *Chlorella*. If photoinhibition were just an unavoidable consequence of PS II turnover, equal turnover rates should induce equal loss in PS II quantum yield. It will be shown that the damaging effect is distinctly larger with blue light.

## Materials and methods

### Experimental setup

**Fig. 1:**
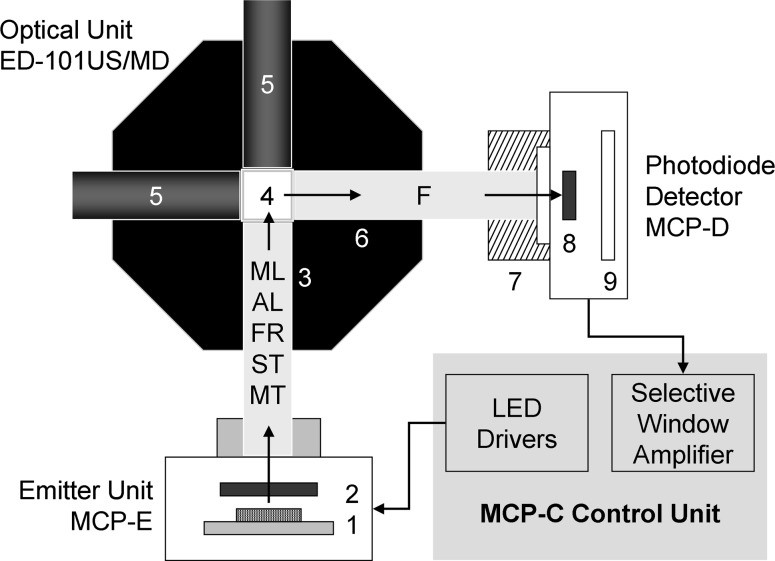
Block diagram of the multi-color-PAM set-up for measurements with suspensions using the optical unit ED-101US/MD (see text for explanations)

The experiments were carried out with a first prototype of a multi-color-PAM chlorophyll fluorometer developed by the authors, which recently has become commercially available (Heinz Walz GmbH, Germany). This device is based on a chip on board (COB) light-emitting diode (LED) array consisting of 60 Power-LED chips mounted on a 10 × 10 mm area, featuring a total of eight different colors, which serve for pulse-modulated ML, AL, FR light, ST pulses, and MT pulses, equivalent to SP. Figure 1 shows a block diagram of the experimental setup. The emitter–detector units are mounted on an Optical Unit with four light-ports (ED-101US/MD), essentially identical to the one introduced for the XE-PAM and phyto-PAM chlorophyll fluorometers (Kolbowski and Schreiber 1995; Schreiber et al. 1993).

Light emission by the multi-color LED array (1) is controlled by separate LED drivers for the various light qualities, which are triggered with 2.5-μs time resolution under firmware/software control. The light passes a short-pass dichroic filter (<640 nm) (2) before it enters a 10 × 10 mm *Perspex* rod (3) that guides it to the 10 × 10 mm glass cuvette (4), mixing the various light qualities by multiple reflections. The suspension within the cuvette (4) is continuously stirred with the help of a small magnetic “flea.” Push-in rods with mirror front faces (5) are inserted at 90° and 180° angles to incident light, thus increasing both the effective light intensities and the amount of fluorescence picked up by a 10 × 10 mm *Perspex* rod (6) at 90° angle to incident light. The fluorescence (*F*) passes a long-pass glass-filter (>650 nm, normally 3 mm RG665) (7), which absorbs scattered incident light, so that only fluorescence reaches the 10 × 10 mm photodiode detector (8). The pulse-modulated fluorescence signal selectively is amplified by a pulse-preamplifier (9) within the detector-unit and then further processed by a special selective-window amplifier within the main control unit.

For standard fluorescence measurements, pulse-modulated ML with peak-wavelengths at 440, 480, 540, 590, and 625 nm is provided (for special applications, not dealt with in this communication, also 400 or 365 nm ML is available). ML pulses, displaying a width of 1 μs, can be applied at wide ranges of pulse intensities (20 settings) and frequencies (10–100,000 Hz), so that time-integrated intensities may differ by a factor of 2 × 10^5^, reaching from virtual darkness to almost saturating light (depending on color and investigated organism).

A separate set of otherwise identical LED-chips with peak-wavelengths at 440, 480, 540, 590, and 625 nm serves for actinic illumination (AL, ST, MT, or SP), supplemented with a white Power-LED (420–645 nm). The latter particularly contributes to saturating multi-color ST. In addition, for preferential excitation of photosystem I (PS I), the LED array features a 725 nm (FR) Power-LED, which is mounted such that the FR can enter the *Perspex* rod (3) without being blocked by the short-pass filter (2).

ST pulses can be applied either with single colors (normally non-saturating) or all colors simultaneously (generally saturating). The “ST pulse intensity,” is adjusted via the width that can be set between 2.5 and 50 μs. Pulse current is always maximal for ST pulses. In contrast, MT pulses or SPs can be applied using single colors only, with the intensity being adjusted via pulse currents (20 settings). While MT pulses and SPs, employing the same LED drivers, optically are fully equivalent, they serve different functions. MT pulses can be triggered with 2.5-μs resolution by preprogrammed Fast Trigger files (possible widths ranging from 2.5 μs to 800 ms) for measurements of fast induction or relaxation kinetics. On the other hand, SP specifically serve for determination of *F*_m_ and $F^{\prime}_{\text{m}}$ in SP quenching analysis (see van Kooten and Snel 1990; Schreiber 2004 for nomenclature). Different SP intensities can be set for *F*_m_ and $F^{\prime}_{\text{m}}$ determination (default settings 3 and 10, respectively), as distinctly less intensity is required to saturate the PS II acceptor side after dark-adaptation than in the illuminated state, when the PS I acceptor side is light activated.

### Measurements of fluorescence yield

The fluorescence yield of a dark-adapted sample, *F*_o_, generally is measured using low frequency (10–50 Hz) of pulse-modulated ML corresponding to photon fluence rates ≪ 1 μmol/(m^2^ s), so that no accumulation of reduced photosystem II (PS II) acceptors can occur. In principle, the integrated intensity of the ML can be sufficiently low (at still satisfactory signal/noise ratio) that closure of so-called inactive PS II (Lavergne and Leci 1993) is avoided. In most experiments, however, FR background light is applied to establish reproducible control conditions in terms of an oxidized plastoquinone (PQ) pool and state 1 (Mullineaux and Emlyn-Jones 2005). FR preillumination results in a rapid small fluorescence increase (about 10 % of *F*_o_) due to the response of “inactive PS II” and a more or less pronounced slow rise of *F*_o_ (*t*_1/2_ in the order of 5 min) reflecting a state 2-state 1 shift (depending on type of cells, temperature, etc.).

The fluorescence yield of an illuminated sample, *F*, normally is measured at substantially higher frequency of pulse-modulated ML (measuring light frequency, MF, 1–100 kHz) than in the case of *F*_o_, with correspondingly enhanced signal/noise ratio and time resolution. Consequently, ML normally contributes significantly to overall actinic intensity, which is accounted for in the PAR value indicated by the user software (see below). In the experiments described in this communication, photons of ML and AL/MT/ST are fully equivalent, as the same colors (batches of LED-chips) were used for all of them.

Slow changes of fluorescence yield were measured in the SP-analysis mode of the software program (PamWin-3). Fluorescence yields *F*_m_ and $F^{\prime}_{\text{m}}$ were measured with 300 ms SP width. Based on the measured values of *F*_o_, *F*_m_, *F*, and $F^{\prime}_{\text{m}}$ the PamWin-3 program automatically calculates maximal and effective PS II quantum yields, *F*_v_/*F*_m_, and Y(II), respectively, as well as various other derived fluorescence parameters (Klughammer and Schreiber 2008; Kramer et al. 2004; van Kooten and Snel 1990).

Light response curves (LC) of relative ETR (rel.ETR) were recorded with the help of Light Curve Program files (lcp-files) programmed for the different colors of light. In general, the same colors were used for ML and AL. Step width at each intensity setting was 3 min. The low-intensity steps were covered by ML at high settings of pulse-frequency. Before start of the LC, samples were dark-adapted for 30 min in the presence of weak FR background light (minimal setting 1) and *O–I*_1_ rise curves were recorded for assessment of Sigma(II)_λ_, the absorption cross section of PS II (see below).

Dark–light–dark induction/recovery curves were measured under the control of Script-file programmed for this purpose. With the help of Script-files, practically all commands that can be carried out manually, can also be programmed with defined time steps between consecutive commands, for fully automated recording. In this way, the experiments were carried out with high reproducibility. Fresh samples were used for each run, which were dark-adapted for 15 min in the presence of weak FR light that was applied throughout the experiment. Identical cell densities were adjusted via identical *F*_o_ signals measured with 440 nm ML at fixed settings of ML-intensity and Gain. When another color of light was used for the actual measurement of light-induced changes, after adjustment of cell densities equal *F*_o_ levels were adjusted via the settings of ML-intensity and Gain, with fine adjustment via the distance between cuvette and photodiode detector (see Fig. 1).

### Measurement of light intensity and PAR-lists

The photon fluence rate (or quantum flux density) of PAR was measured with a calibrated quantum sensor (US-SQS/WB, Walz), featuring a 3.7-mm diffusing sphere, mounted in the center of the cuvette filled with water. This sensor is connected via an amplifier box directly to the External Sensor input of the MCP-C Control Unit. The PamWin software provides a routine for automated measurements of ML, AL, and MT/SP intensities of all the colors at 20 settings each. The measured values are saved in the so-called PAR-lists, on which calculation of PAR-dependent parameters is based. PAR and fluorescence measurements were carried out under close to identical optical conditions. Detailed knowledge of incident PAR (in units of μmol/(m^2^ s)) effective within the suspension during illumination with different colors of ML, AL, and MT/SP is essential for quantitative analysis of the light responses. As all measurements were carried out at low cell densities, also transmitted light reflected back into the sample (see Fig. 1) contributed significantly to overall intensity, which was accounted for using the spherical sensor. While strictly speaking in this case the term photosynthetic photon fluence rate (PPFR) may apply (Braslavsky 2007), for the sake of simplicity in PAM applications the abbreviation PAR has been used.

### Measurements of fast kinetic responses

**Fig. 2:**
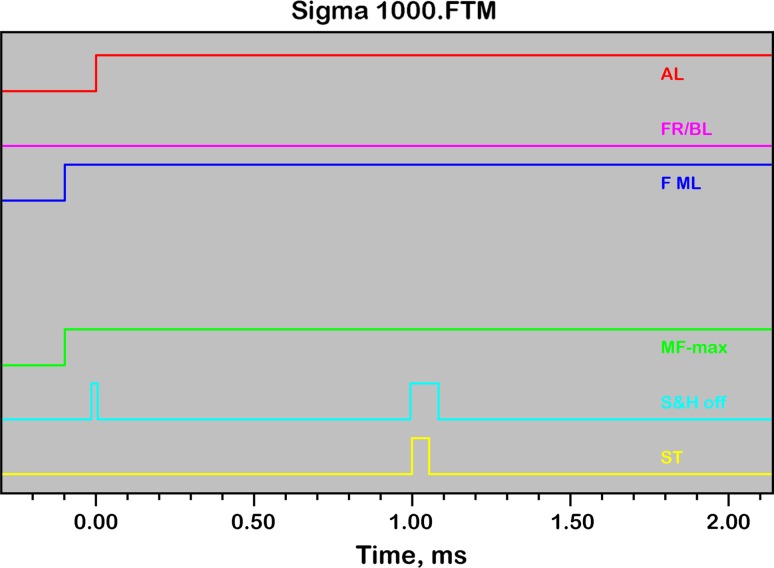
Screenshot of the Fast Trigger pattern programmed for measurements of *O*–*I*_1_ rise kinetics. On–off times of six different triggers are depicted: *AL* actinic light, *FR*/*BL*, far-red or blue light pretrigger PS I illumination (not active, as FR background instead), *F ML* pulse-modulated fluorescence ML, *MF*_*max*_ maximal ML frequency, 100 or 200 kHz, *S&H off* Sample&Hold off gating period of amplifier, *ST* single-turnover pulse (see text for further explanations)

Fast kinetic responses were measured under the control of so-called Fast Trigger files, which were programmed such that rapid changes of light intensity, as occurring upon AL-on/off, MT-on/off, or during an ST pulse, do not affect the pulse-modulated signal. The Sample-and-Hold off (S&H off) Trigger is essential for avoiding artifacts induced by rapid changes of non-modulated light. During the S&H off time the sample-and-hold amplifier, which processes the pulse-modulated signal, is “gated” (i.e., switched off). Figure 2 shows a screenshot of the Fast Trigger pattern of the file Sigma1000.FTM that was programmed for reproducible measurements of the so-called *O–I*_1_ rise kinetics (for nomenclature see Schreiber 2004) and determination of the sample- and wavelength-dependent absorption cross section of PS II, Sigma(II)_λ_, which play a central role in the present report.

The Fast Trigger pattern of the file Sigma1000.FTM displayed in Fig. 2 may serve to outline some points essential for optimal measurements of the *O–I*_1_ rise kinetics:

1. The pulse-modulated fluorescence ML is switched on only 100 μs before onset of AL to minimize the fluorescence rise induced by the ML and, hence, to allow use of relatively high ML-intensity setting for the sake of a high signal/noise ratio.
2. Maximal measuring pulse-frequency (MF_max_) is triggered simultaneously with ML-on. The default setting of MF_max_ = 100 kHz provides sufficient time resolution for reliable assessment of the *O–I*_1_ kinetics with time constants in the order of 200 μs.
3. AL is triggered at time −5 μs to take account of a small time delay between switching of the AL-LED-driver and AL-on.
4. The amplifier “gating” (S&H off) is triggered on for 15 μs for AL-on (from −10 to 5 μs) and for 80 μs for the 50 μs ST pulse (from 995 to 1,075 μs).

Consecutive measurements of *O–I*_1_ rise kinetics driven by strong 440-, 480-, 540-, 590-, and 625-nm light of the same sample were preprogrammed in special Script-files for *Chlorella* and *Synechocystis* with 10-s dark-time between measurements. For each color, ML-intensity/Gain settings were programmed to give approximately equal *F*_o_ values. AL/MT-intensity settings were programmed such that for the investigated organism the initial rise curves displayed similar slopes with all the colors.

### Analysis of *O*–*I*_1_ rise kinetics

The kinetics of the *O*–*I*_1_ fluorescence rise were analyzed with the help of a dedicated fitting routine developed for determination of the wavelength-dependent absorption cross section of PS II, here called Sigma(II)_λ_. Fitting is based on the reversible radical pair model of PS II originally described by Lavergne and Trissl (1995) that was extended to take account of Q_A_^−^-reoxidation (Klughammer C, Kolbowski J and Schreiber U, in preparation). Variable parameters in this model, fitted by the PamWin-3 program, are:

*J*: Sigmoidicity parameter, which is related to Joliot’s connectivity parameter, *p*, via the equation *J* = *p*/(1 – *p*)
Tau: Time constant of light-driven reduction of Q_A_ (by AL or MT pulse), corresponding to the inverse of the rate constant of PS II turnover, *k*(II)
Tau(reox): Time constant of Q_A_^−^-reoxidation.

Directly measured parameters are the *F*_o_ and *I*_1_-levels, which define the total range of ∆*F* that can be induced by a saturating ST flash (ST pulse) in the presence of an oxidized PQ-pool. The fitted parameters refer to the kinetics of Q_A_-reduction, i.e., the increase of (1 − *q*), where *q* represents the fraction of open PS II reaction centers. The relationship between variable fluorescence and (1 − *q*) is described by the equation (*F* – *F*_o_)/(*I*_1_ – *F*_o_) = (1 – *q*)/(1 + *J* *q*) in analogy to an equation derived by Lavergne and Trissl (1995).

The *O*–*I*_1_ curves measured with the five different colors were fitted together with the restriction of common values of *J* and Tau(reox), as these parameters are unlikely to depend on the color of light. Calculation of Sigma(II)_λ_ by the multi-color-PAM-software is based on the fitted value of the time constant Tau and the value of incident PAR, using the following general equation:

1 $$ {\text{Sigma}}({\text{II}})_{\lambda } = \frac{{k({\text{II}})}}{{L \cdot {\text{PAR}}}} = \frac{1}{{\tau \cdot L \cdot {\text{PAR}}}}, $$

where *k*(II) is the rate constant of PS II turnover and Tau the time constant of Q_A_-reduction during the *O*–*I*_1_ rise, *L* is Avogadro’s constant, PAR is the photon fluence rate of the light driving the *O*–*I*_1_ rise and Sigma(II)_λ_ the wavelength- and sample-dependent absorption cross section of PS II (for further explanations, see “Results and interpretation” section).

### Measurement of absorptance

Sample absorptance was measured using the same Optical Unit ED-101US/MD as for fluorescence measurements (see Fig. 1), but with the detector-unit MCP-D being moved from the 90° position (relative to the emitter-unit) to the 180° position. The long-pass filter in front of the detector was exchanged against suitable neutral density filters and pin-hole diaphragms, so that pulse-modulated transmittance signals could be measured both with the suspension medium as such, *I*_medium_, and with the suspension medium containing *Chlorella* or *Synechocystis*, *I*_sample_. The absorptance a (=1 − transmittance) was calculated as *a* = 1 – *I*_sample_/*I*_medium_. With the given optical geometry almost all light entering the 10 × 10 mm cuvette via the emitter-perspex-rod is picked up by the detector-perspex-rod, unless absorbed by the sample.

### Photosynthetic organisms and sample preparation

Experiments were carried out with dilute suspensions of green unicellular algae *Chlorella vulgaris* and cyanobacteria *Synechocystis* PCC 6803. *Chlorella* was cultured in natural day light (north window) at 20–40 μmol/(m^2^ s) and room temperature (25 °C) in an inorganic medium (Pirson and Ruppel 1962) under ambient air. *Synechocystis* was grown photoautotrophically in artificial light (tungsten) at 30  μmol/(m^2^ s) and 30 °C in Allen’s (1968) medium under ambient air. Both cultures were shaken manually at least four times per day. Cultures were frequently diluted so that chlorophyll content did not exceed 5–10 mg/L. Experiments were carried out at room temperature with diluted suspensions at 200–300 μg/L, as determined with a calibrated WATER-PAM chlorophyll fluorometer (Walz).

For sample preparation the cuvette was first filled with 1.4 mL of culture medium and then stock suspension was added dropwise to the stirred sample until signals corresponding to 200–300 μg/L were reached. Settings of ML-intensity and Gain were adjusted to obtain *F*_o_ values of about 2 and 3 V in the case of *Chlorella* and *Synechocystis*, respectively.

## Results and interpretation

### Wavelength dependence of normalized *F*_o_/PAR and absorptance

The most important parameters determining the intensity of chlorophyll fluorescence are (1) quantum flux density of incident photosynthetically active light (PAR), (2) spectral composition of the incident light, (3) absorption spectrum of the photosynthetic organism, (4) cell density/chlorophyll content and (5) state of PS II in terms of reduction of the primary acceptor Q_A_ and down-regulation by non-photochemical quenching (NPQ). The effect of the last parameter can be considered constant, when samples are dark-acclimated in the presence of weak FR light that oxidizes the PQ-pool resulting in the so-called state 1, provided the intensity of the pulse-modulated ML is sufficiently low, so that it does not change the state of PS II. When this prerequisite is fulfilled, at constant PAR of incident ML and chlorophyll content of the sample, the wavelength dependence of the fluorescence signal reflects the overlapping integral between the spectrum of the incident light and the absorption spectrum of the photosynthetic pigments that transfer the excitation energy to PS II. When narrow band excitation is used, as is the case with standard spectrofluorometers, fluorescence intensity per incident quanta measured as a function of wavelength results in an excitation spectrum. The multi-color-PAM provides relatively broad-band light (half-band width 15–25 nm) peaking at 440, 480, 540, 590, and 625 nm, resulting in a coarse five-point excitation spectrum.

**Fig. 3:**
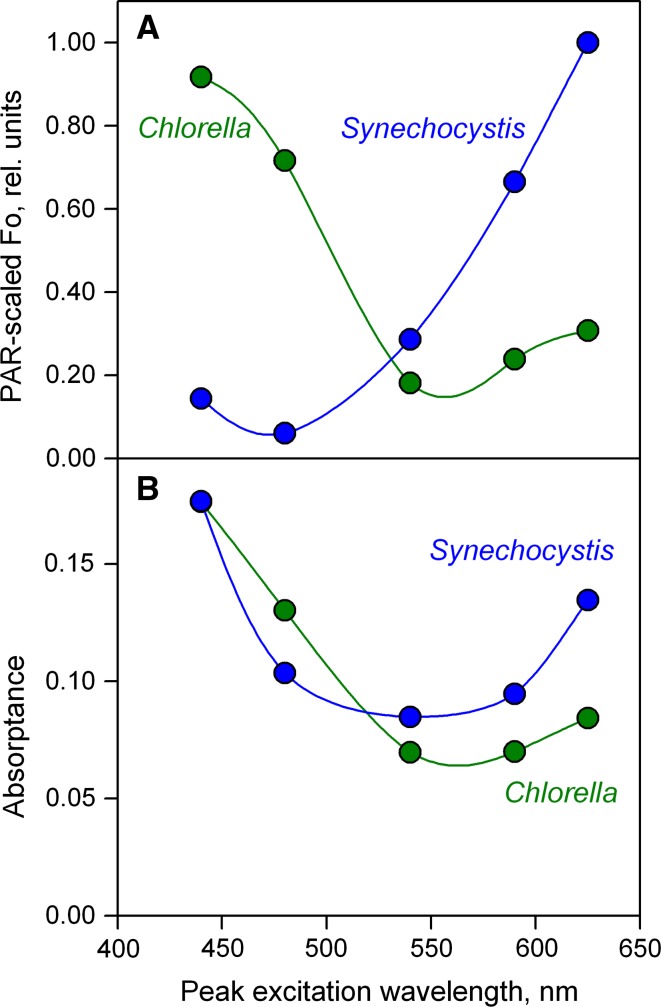
Comparison of PAR-scaled *F*_o_ and absorptance in dilute suspensions of *Chlorella* and *Synechocystis* as a function of the color of the pulse-modulated ML. Cell densities of the two suspensions were adjusted to give the same absorptance at 440 nm. **A** Normalized *F*_o_/PAR versus peak wavelength of the ML. The data were normalized to unity at maximal relative *F*_o_/PAR, i.e., for 625 nm with *Synechocystis*. **B** Absorptance in the same suspensions plotted vs peak wavelength of the ML

In Fig. 3A and Table 1, the *F*_o_ values measured with 440, 480, 540, 590, and 625 nm ML in dilute suspensions of green algae (*Chlorella vulgaris*) and cyanobacteria (*Synechocystis* PCC 6403) are compared using identical settings of Gain (signal amplification). The cell densities in the two suspensions were adjusted to give the same absorptance at 440 nm (see “Materials and methods”). At the applied ML-intensity settings the intensities of the incident PAR generally were too low to induce any fluorescence increase beyond *F*_o_ (even with respect to “inactive PS II”). Division of the measured *F*_o_ values by the incident PAR (derived from instrument specific PAR-lists) and normalization results in the so-called PAR-scaled *F*_o_ values, equivalent to *F*_o_ values as would be measured with equal photon fluence rates at different wavelengths. PAR-scaled *F*_o_ plotted against the peak-wavelengths corresponds to a fluorescence excitation spectrum (see Fig. 3A). The *F*_o_/PAR data were normalized to 1 relative unit at the maximal signal value, which was observed with *Synechocystis* using 625-nm excitation.

**Table 1:** Comparison of *F*_o_ and *F*_o_/PAR of dilute suspensions of *Chlorella* and *Synechocystis* measured with five different colors at identical settings of ML-intensity and minimal pulse-frequency

Parameter					
Peak wavelength (nm)	440	480	540	590	625
Incident PAR (μmol/(m^2^ s))	0.0234	0.0309	0.0201	0.0099	0.0159
Incident PAR (rel. units)	75.7	100.0	65.2	32.0	51.5
*F*_o_(*Chlorella*)_λ_ (V)	2.294	2.366	0.389	0.252	0.522
*F*_o_(*Chlorella*)_λ_/PAR (rel. units)	0.917	0.716	0.181	0.238	0.307
*F*_o_(*Synechocystis*)_λ_ (V)	0.359	0.198	0.616	0.703	1.702
*F*_o_(*Synechocystis*)_λ_/PAR (rel. units)	0.143	0.060	0.286	0.665	1.000

The *F*_o_/PAR values were normalized to give 1 rel. unit at 625 nm with *Synechocystis*, where the maximal signal was obtained

As may be expected in view of the differences in photosynthetic pigments serving PS II, the wavelength dependence of dark-fluorescence yield, *F*_o_, differs considerably between *Chlorella* and *Synechocystis*. Somewhat unexpectedly, despite the identical absorptance at 440 nm, i.e., although the same fraction of incident 440 nm quanta is absorbed in the *Chlorella* and *Synechocystis* suspensions, the *F*_o_(*Chlorella*)_440_ exceeds the *F*_o_(*Synechocystis*)_440_ by a factor of 2.294/0.359 = 6.4 (see Table 1). Absorption at 440 nm is dominated by Chl *a* and, hence, Chl *a* concentration should be close to identical in the two samples. The large difference in *F*_o_/PAR values may be explained by a higher fluorescence yield of Chl *a* (PS II) as compared to Chl *a* (PS I) and to a higher PS I/PS II ratio in *Synechocystis* than in *Chlorella*. In contrast, when with the same samples 625 nm ML is used, the *F*_o_(*Synechocystis*)_625_ exceeds the *F*_o_(*Chlorella*)_625_ by a factor of 1.702/0.522 = 3.3. In *Synechocystis*, the peak of absorption by phycocyanin is at 625 nm, whereas in *Chlorella* this wavelength is at some distance from the main Chl *a*/*b* absorption peaks.

The *F*_o_/PAR plots of *Chlorella* and *Synechocystis* in Fig. 3A can be compared with the corresponding absorptance spectra in Fig. 3B, measured under identical optical conditions (see “Materials and methods”). While the spectra of *F*_o_/PAR and absorptance resemble each other with *Chlorella*, they differ substantially in the case of *Synechocystis*. PS I-specific absorption is higher in *Synechocystis* than in *Chlorella* due to a higher PS I/PS II ratio. Also, the more PS I-specific absorption differs from PS II-specific absorption, the more the overall absorptance spectrum will differ from the *F*_o_/PAR spectrum. Therefore, *F*_o_/PAR spectra can provide more specific information on PS II absorption, than absorptance spectra.

This finding appears important in connection with attempts to model primary production on the basis of measurements of chlorophyll fluorescence and calculation of photosynthetic absorbed radiation *Q*_phar_ from surface irradiance and Chl *a*-specific absorption in successive water layers (Gilbert et al. 2000a, b; Jakob et al. 2005). Obviously, the wavelength dependencies of *Q*_phar_ and of the rate of PS II-specific quanta absorption can differ substantially. PS II charge-separation rate is decisive for the overall rate of photosynthetic electron transport.

While PAR-scaled *F*_o_ may qualify as a satisfactory proxy for estimating the *relative* extent of PS II excitation by the five different colors of light provided by the multi-color-PAM, it does not carry information on the *absolute* rates. As will be shown below, such information can be derived from measurements of the wavelength-dependent *O*–*I*_1_ rise kinetics.

### Wavelength dependence of relative electron transport rate in *Chlorella*

The light response of photosynthetic organisms can be routinely analyzed with the help of fluorescence-based light curves (LCs), consisting of a number of illumination steps using increasing intensities of PAR. The longer the illumination steps the more the fluorescence-based LCs approach classical *P*–*I* curves (photosynthesis vs. irradiance curves), where steady state is reached within each PAR-step, before photosynthetic rate is evaluated. PAM fluorometers allow more or less rapid LC-recordings of various fluorescence-derived parameters, like the effective PS II quantum yield, Y(II), and relative electron transport rate, rel.ETR (see, e.g., Herlory et al. 2007; Ralph and Gademann 2005; Rascher et al. 2000; Schreiber et al. 1994). For LCs with illumination times too short to reach steady state, the term rapid LCs (RLCs) was coined (Schreiber et al. 1997).

Rel.ETR as a fluorescence-derived parameter originally was introduced for PAM-measurements with leaves (Schreiber et al. 1994)

2 $$ {\text{rel}} . {\text{ETR}} = {\text{Y}}({\text{II}}) \cdot {\text{PAR}} \cdot {\text{ETR-factor}} $$

The ETR-factor is supposed to account for the fraction of overall incident PAR that is absorbed within PS II. In most published studies, however, no attempt has been made to determine the ETR-factor, which simply has been assumed to correspond to that of a “model leaf,” with 50 % of the PAR being distributed to PS II and 84 % of the PAR being absorbed by photosynthetic pigments in a standard leaf (Björkman and Demmig 1987), so that normally a default ETR-factor of 0.42 is applied.

Without detailed knowledge of the true PS II-specific absorbance, ETR can give a rough estimate only of relative photosynthetic electron transport rate. In the case of dilute algae suspensions, where a minor part of overall incident radiation is absorbed, normally rel.ETR is just treated as an intrinsic parameter of the relative rate of PS II turnover. With this kind of approach, rel.ETR is independent of Chl content, just like Y(II), from which it is derived and, hence, essentially describes the relative frequency of charge-separation at PS II reaction centers. LCs of rel.ETR defined in this way provide useful information, as long as the performance of a particular organism is studied using a fixed color of light, as is the case with standard PAM fluorometers. For this purpose, standard PAM-software provides routines for fitting the LC-parameters α, rel.ETR_max_, and *I*_*k*_ using models developed by Eilers and Peeters (1988) or Platt et al. (1980). The parameter α relates to the maximal PS II quantum yield (initial slope of LC). Rel.ETR_max_ is a measure of maximal relative rate and *I*_*k*_ relates to the PAR at which light saturation sets in (defined by ETR_max_/α). For example, diurnal changes in rel.ETR_max_ (measured with the same sample in its natural environment) provide valuable information on changes of photosynthetic capacity due to light-dependent enzyme regulation and down-regulation of PS II upon exposure to excess light (Ralph et al. 1999).

**Fig. 4:**
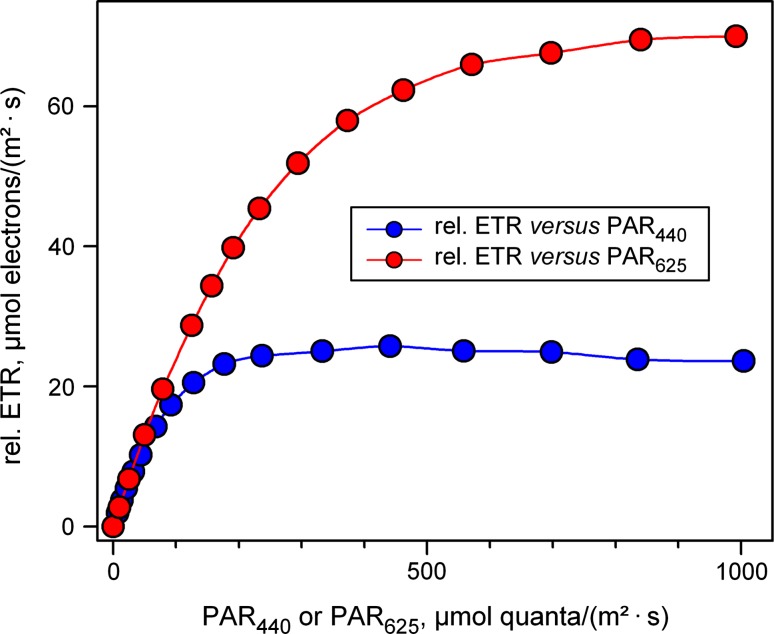
LC of rel.ETR measured with a dilute suspension of *Chlorella* (300 μg Chl/L) using 440- and 625-nm light. Ignoring information on the fraction of incident light absorbed by PS II, a default ETR-factor of 0.42 was applied (see text for explanation and Fig. 8 for comparison). Illumination time at each intensity-setting was 3 min

While most PAM fluorometers so far have been providing just one color of ML (red or blue) and AL (normally white, red or blue), with the new multi-color-PAM light response curves of the same sample can be recorded using different colors. As expected, in this case substantial differences in LC-parameters are revealed, when a default value of 0.42 is applied as ETR-factor. In Fig. 4, LCs of rel.ETR in *Chlorella* with 3-min illumination steps using 440- and 625-nm light are compared.

With 440-nm light the rel.ETR LC saturates at much lower PAR than with 625-nm light and the rel.ETR_max_ measured with 440 nm is much lower than when measured with 625 nm. Furthermore, with 440 nm after reaching maximal values of rel.ETR, there is some decline of rel.ETR, which is not apparent with 625-nm illumination. The decline of rel.ETR is likely to reflect photoinhibition and, hence, the observed differences between 440- and 625-nm illumination seem to agree with previous findings that blue light is more effective than red light in causing photoinhibition. At this stage, however, it would be premature to interpret these data as evidence for the two-step hypothesis of photoinhibition (see “Introduction”), with the rate-limiting step consisting of blue-light-induced damage of the OEC. Obviously, 440-nm photons are much better absorbed by PS II than 625-nm photons, so that the data also agree with the notion that the extent of photoinhibition increases with the rate of PS II turnover. The decisive question is whether more photoinhibition is also observed when the same flux density of *PS II-absorbed* 440- and 625-nm photons is applied. This aspect will be further investigated below (see Figs. 8, 9).

**Fig. 5:**
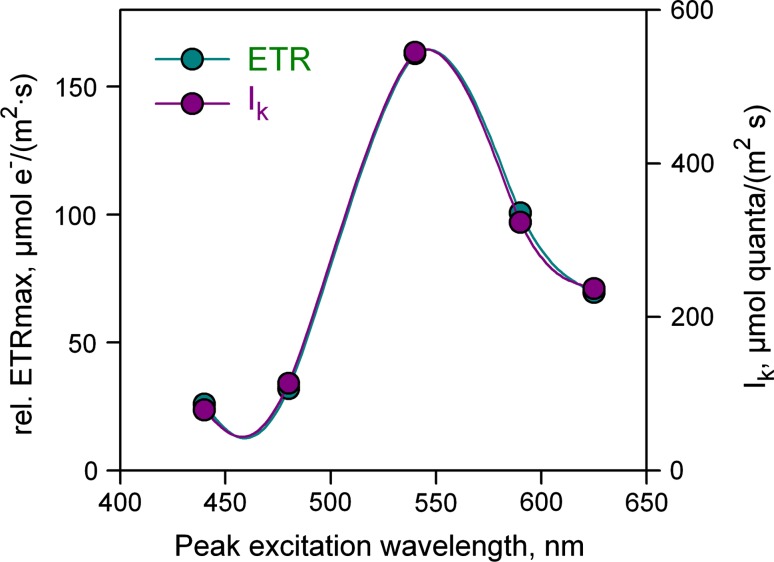
Rel.ETR_max_ and *I*_*k*_ values of *Chlorella* plotted against the peak wavelength of the AL. Rel.ETR LCs were measured with same *Chlorella* sample using different AL colors and a default ETR-factor of 0.42. Parameters were fitted by a PamWin-3 routine based on the model of Eilers and Peeters (1988)

In Fig. 5, the wavelength-dependent LC-parameters for all five colors are displayed, as derived from consecutive measurements of rel.ETR LC at 440, 480, 540, 590, and 625 nm, with consequent software-assisted fitting of the various LC-parameters according to the model of Eilers and Peeters (1988).

These data show that the same quantum flux density of differently colored light within the range of “PAR” can have vastly different effects, not only between differently pigmented organisms but also within the same organism. Notably, in *Chlorella* there are even considerable differences between the two types of blue light (440 and 480 nm).

Rel.ETR_max_ and *I*_*k*_ display almost identical wavelength dependency, in the case of *Chlorella* with peak and minimal values at 540 and 440 nm, respectively. The ETR_max_ and *I*_*k*_ spectra resemble inverse *F*_o_/PAR spectra (see Fig. 2). It should be kept in mind, however, that PS I contributes to *F*_o_, and that rel.ETR_max_ as well as *I*_*k*_ not only depend on PS II but also on PS I activity.

The multi-color-PAM has opened the way for detailed studies of electron transport as a function of the color of light in photosynthetic organisms with largely different pigment compositions. From the above data it is obvious that for such measurements, either a wavelength- and sample-dependent ETR-factor has to be defined or the quantum flux density of PAR has to be replaced by a PS II-specific quantum flux rate, PAR(II). The latter approach is advantageous, as it results in determination of an absolute rate, independent of chlorophyll content. It requires information on the wavelength- and sample-dependent functional absorption cross section of PS II, Sigma(II)_λ_.

### PAR and the wavelength-dependent functional absorption cross section of PS II, Sigma(II)_λ_

Usually, PAR is defined for wavelengths between 400 and 700 nm (Sakshaug et al. 1997) in units of μmol/(m^2^ s). It is determined with calibrated quantum sensors, which measure the overall flux density of incident photons, without making any distinction between photons of different colors, as long as their wavelengths fall into the 400–700-nm PAR range.

Hence, the actual extent of PAR-absorption (whether by PS II or PS I or any other colored constituents) by the photosynthetically active sample normally is *not* taken into account. While this kind of approach has been widely accepted in the study of leaves, which display relatively flat absorbance spectra and absorb most of the incident light, it is not feasible with dilute suspensions of unicellular algae and cyanobacteria, where PS II excitation by light of different wavelengths may vary by an order of magnitude and only a fraction of the incident light is absorbed.

Rappaport et al. (2007) recently pointed out that the “most commonly used unit for light intensity… μmol of photons s^−1^ m^−2^ … has little experimental value since it cannot reliably be translated into a photochemical rate without knowing the absorbance of the sample, which is rarely the case”. Further, the authors note that “there is… a real need for a more relevant unit which should be the number of electrons transferred per unit time and per PS II reaction center.” Rappaport et al. (2007) determined the rate of PS II turnover via the rate constant of the fluorescence rise induced in the presence of DCMU. As will be outlined below, for quantitative work with the multi-color-PAM, e.g., analysis of light response curves, we prefer to translate the quantum flux density (or photon fluence rate) of PAR into a photochemical rate on the basis of information on PS II absorbance of the sample, obtained via measurements of rapid induction kinetics *in the absence* of DCMU.

Obviously, the PAR information has to be complemented with information on the PS II efficiency of the applied PAR with respect to a given sample. Such information is contained in the wavelength-dependent functional absorption cross section of PS II, the Sigma(II)_**λ**_, which depends on both the spectral composition of the applied irradiance (i.e., the AL-color) and the PS II absorption properties of the investigated sample. The value of Sigma(II)_λ_ can be derived from the initial rise of fluorescence yield upon onset of saturating light intensity, which directly reflects the rate at which PS II centers are closed. The rate of charge-separation of open PS II centers, *k*(II), matches the rate with which photons are absorbed by PS II, which may be defined as PAR(II) (see below). In order to account for the overlapping re-opening of PS II centers by secondary electron transport (reoxidation of Q_A_^−^ by Q_B_), either a PS II inhibitor-like DCMU has to be added, which is not feasible for in vivo studies, or PAR(II) has to be extremely high, so that the reoxidation can be ignored (Koblizek et al. 2001; Kolber et al. 1998; Nedbal et al. 1999), or the rise kinetics have to be corrected for the reoxidation rate.

The last approach is applied with the multi-color-PAM, which is outlined in detail in a separate publication (Klughammer C, Kolbowski J and Schreiber U, in preparation). Here, just one original measurement with a dilute suspension of *Chlorella* using 440-nm light is presented, which may serve to outline the principle of the approach.

**Fig. 6:**
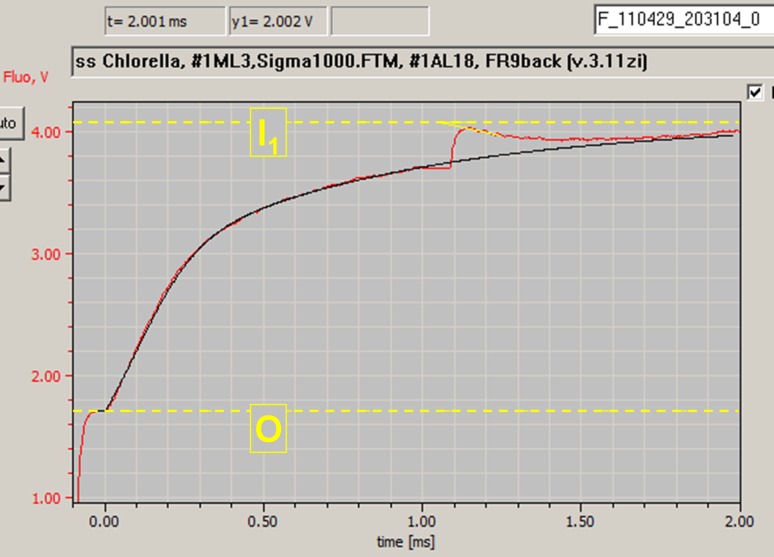
Initial increase of fluorescence yield (*O*–*I*_1_ rise) in a dilute suspension of *Chlorella* (300 μg Chl/L) induced by 440-nm AL with 2,131 μmol quanta/(m^2^ s) in presence of FR background light. *Dashed yellow lines* indicate *F*_o_-level (O), assessed during a 50-μs period preceding onset of AL at time zero, and the *I*_1_-level that is determined with the help of a saturating single-turnover pulse (ST) triggered 1 ms after onset of AL (see Fig. 2 for the Fast Kinetics trigger pattern). The slope of the relaxation kinetics is extrapolated to the end of the 50-μs ST. The *black line* represents the *O*–*I*_1_ fit curve based on a PS II model which incorporates energy transfer between PS II units and reoxidation of the primary PS II acceptor Q_A_ (see text)

Figure 6 shows the initial part of the increase of fluorescence yield induced by strong AL (in PAM-literature called *O*–*I*_1_ rise). The *O*–*I*_1_ rise basically corresponds to the *O*–*J* phase of the polyphasic OJIP kinetics that have been described in detail by Strasser and co-workers (for reviews see Strasser et al. 2004; Stirbet and Govindjee 2011). There are, however, essential differences in the measuring techniques and definitions of the characteristic fluorescence levels *I*_1_ and *J*, which argue for different nomenclatures. The multi-color-PAM allows to use the so-called Fast Trigger Files (see “Materials and methods”) for routine measurements of fast kinetics. In the example of Fig. 6, the pulse-modulated ML was triggered with 100 kHz pulse-frequency at 100 μs before onset of 440 nm AL. At 1 ms after onset of AL, a saturating 50-μs multi-color ST pulse was applied. The ST pulse closes PS II reaction centers transiently, so that the *I*_1_-level of fluorescence yield can be determined by extrapolation to 1,050 μs. *I*_1_ corresponds to the maximal fluorescence yield that can be reached in the presence of an oxidized PQ-pool (for apparent PQ-quenching see Samson et al. 1999; Schreiber 2004). Weak FR background light or short FR-preillumination is routinely applied to assure a fully oxidized PQ-pool. This aspect is particularly important in the study of algae and cyanobacteria, where depending on conditions the PQ-pool becomes more or less reduced in the dark via NADPH-dehydrogenase activity, resulting in more or less transition into state 2. Furthermore, FR-preillumination minimizes the contribution of “inactive PS II” to the *O*–*I*_1_ kinetics.

At a first approximation, assuming that the AL-driven increase of fluorescence yield is linearly correlated with accumulation of Q_A_^−^, and that the initial rise is negligibly slowed down by Q_A_^−^ reoxidation, the kinetics can be described by a first order reaction, of which the time constant Tau = 1/*k*(II) corresponds to the time for reaching a Q_A_-reduction level of 100(1 − 1/*e*) = 63.2 %. When this approximation is applied to the *O*–*I*_1_ rise of Fig. 6, Tau = 0.379 ms is estimated. A thorough analysis of the *O*–*I*_1_ rise kinetics, however, has to take into account both Q_A_^−^ reoxidation and nonlinearity between ∆*F* and the fraction of reduced *Q*_A_. This can be achieved by a fitting routine we have specially developed for this purpose (see “Materials and methods”).

For the *O*–*I*_1_ rise displayed in Fig. 6, which was driven by 2,131 μmol quanta/(m^2^ s) of 440-nm AL, the following values were estimated by the *O*–*I*_1_ fit routine:

Tau = 0.173 ms, *k*(II) = 1/Tau = 5.78 × 10^3^/s, Tau(reox) = 0.340 ms, *J* = 2.01 (corresponding to *p* = 0.67), Sigma(II)_440_ = 4.51 nm^2^.

Consecutive measurements of *O*–*I*_1_ rise kinetics using 440, 480, 540, 590, and 625 nm with the same sample are facilitated by the so-called Script-files (see “Materials and methods”). The kinetic parameters of all five rise curves can be fitted together. An example of the obtained data for a dilute suspension of *Chlorella* is presented in Table 2, which also shows analogous data for *Synechocystis*.

**Table 2:** Data from consecutive measurements of *O*–*I*_1_ rise kinetics in *Chlorella vulgaris* and *Synechocystis* PCC 6803

Parameter							
Peak wavelength (nm)	*F*_o_ (V)	*I*_1_ (V)	PAR (μmol/(m^2^ s))	*J*	Tau (ms)	Tau(reox) (ms)	Sigma(II) (nm^2^)
*Chlorella vulgaris*							
440	2.199	4.981	1579	2.043	0.231	0.341	4.547
480	2.237	5.198	2160	2.043	0.229	0.341	3.353
540	2.375	5.302	9649	2.043	0.228	0.341	0.756
590	2.293	5.205	6125	2.043	0.238	0.341	1.138
625	2.053	4.710	4426	2.043	0.225	0.341	1.669
*Synechocystis* PCC 6803							
440	3.193	5.243	2679	2.232	0.543	0.521	1.141
480	3.245	4.752	9358	2.232	0.538	0.521	0.330
540	3.273	4.898	1907	2.232	0.537	0.521	1.621
590	3.232	4.943	634	2.232	0.511	0.521	5.123
625	3.265	5.037	382	2.232	0.506	0.521	8.597

Tau values (time constant of Q_A_-reduction) were separately fitted for the five colors, whereas common fits of Tau(reox) (time constant of Q_A_ oxidation) and *J* (connectivity) were applied

The fits of Table 2 were carried out under the assumption that the values of the connectivity parameter, *J*, and of the Q_A_^−^ reoxidation time constant, Tau(reox) are equal for all colors. It may be noted that the values of the Q_A_-reduction time constant, Tau, were similar for all colors, whereas the applied photon flux rates, PAR, were vastly different. For both the organisms the settings of AL and MT pulse intensities on purpose were programmed to induce rise kinetics with similar initial slopes for all colors. At constant Tau the wavelength-dependent absorption cross section is inversely proportional to the applied PAR (for calculation of Sigma(II), see “Materials and methods”), which is always true, independently of the underlying model of PS II primary reactions. Therefore, with this kind of approach, potential errors due to deficiencies in our model are minimized. Obviously, this approach heavily relies on accurate values of PAR within the sample. For this purpose, the multi-color-PAM features detailed PAR-lists (see “Materials and methods”), for measurement of which an automated routine is provided.

**Fig. 7:**
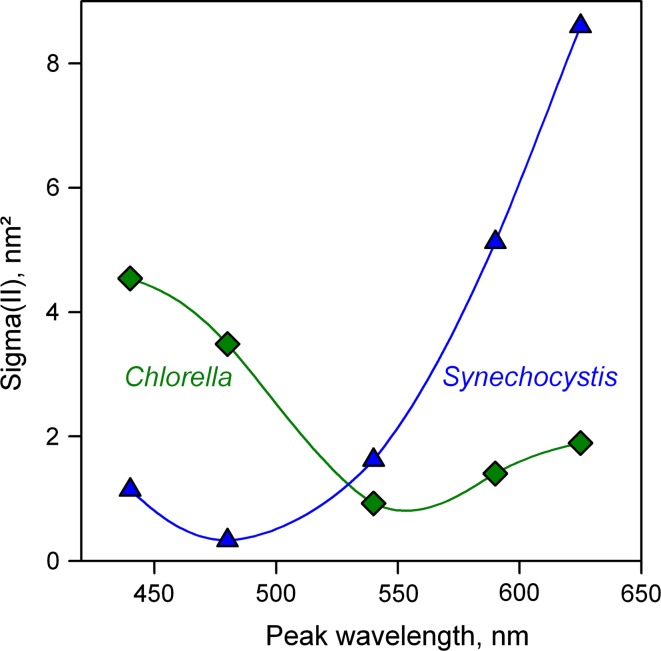
Functional cross section of PS II, Sigma(II) as a function of AL-color in dilute suspensions (300 μg Chl/L) of *Chlorella* and *Synechocystis*, derived from automated measurements of five consecutive *O*–*I*_1_ rise curves each (Script-files Sigma1000Chlor_10.prg and Sigma1000Sycy_10.prg) in the presence of FR background light. Time between consecutive *O*–*I*_1_ measurements, 10 s. Sigma(II) values derived by dedicated PamWin-3 fitting routine (see text and Table 2)

In Fig. 7, plots of Sigma(II)_λ_ as a function of the peak wavelength are presented for *Synechocystis* and *Chlorella*. As expected, these plots resemble fluorescence excitation spectra, similar to the plots of *F*_o_/PAR presented in Fig. 3A. On closer inspection, comparison of the *F*_o_/PAR and Sigma(II)_λ_ spectra reveals that there are significant differences for *Synechocystis* and much less for *Chlorella*. In *Synechocystis*, the ratio of maximal to minimal Sigma(II) (at 625 and 480 nm, respectively) is 26.1, whereas the corresponding ratio of *F*_o_/PAR amounts to 15.5. On the other hand, in *Chlorella* the ratio of maximal to minimal Sigma(II) (at 440 and 540 nm) is 6.0, as compared to 5.1 of the corresponding *F*_o_/PAR. This finding confirms that Sigma(II)_λ_ is a more specific measure of PS II excitation than *F*_o_/PAR. While *F*_o_ may contain more or less non-PS II fluorescence, depending on excitation wavelength and organism, variable fluorescence yield and the rate with which it is induced, are specific for PS II. Another important difference between Sigma(II) and *F*_o_/PAR is that Sigma(II) gives *absolute* information on the functional absorption cross section of PS II, which is independent of Chl content, whereas *F*_o_/PAR is proportional to both Chl content and functional cross section of PS II. Furthermore, *F*_o_/PAR depends on ML-intensity and gain parameters, which have no influence on Sigma(II), as measured with the multi-color-PAM.

### Definition of PAR(II) and ETR(II)

The wavelength-dependent rate, with which photons (or quanta) are absorbed by PSII, is directly reflected in the *k*(II) determined by fitting the *O*–*I*_1_ rise kinetics measured at high PAR under defined control conditions (see text accompanying Fig. 6). There is direct correspondence between the PS II turnover rate, *k*(II), in units of electrons/(PS II s) and the quantum absorption rate at PS II reaction centers in units of quanta/(PS II s). We propose the name PAR(II) for the latter, with the general definition derived from Eq. 1 (see “Materials and methods”)

3 $$ {\text{PAR}}({\text{II}}) = k({\text{II}}) = {\text{Sigma}}({\text{II}})_{\lambda } \cdot L \cdot {\text{PAR}}, $$

where *k*(II) is the rate constant of PS II turnover, Sigma(II)_λ_ is the functional cross section of PS II (in units of nm^2^), *L* is Avogadro’s constant (with the dimension of mol^−1^), PAR is quantum flux density (or photon fluence rate) and PAR(II) is the rate of quantum absorption in PS II, in units of quanta/(PS II s).

In practice, calculation of PAR(II) from PAR is quite simple when Sigma(II)_λ_ is known: the numerical value of PAR (in units of μmol quanta/(m^2^ s)) just has to be multiplied by 0.6022 **×** Sigma(II)_λ_. Hence, once Sigma(II) has been determined for a particular color and sample (via measurement of the *O*–*I*_1_ rise kinetics at a defined high light intensity), PAR(II) can be derived for any other PAR (at constant color and state of the sample), without further measurements of fast kinetics. In the case of *Chlorella*, with Sigma(II)_625_ = 1.669 (see Table 2), PAR(II) practically equals PAR, as 0.6022 **×** 1.669 happens to be very close to unity.

An essential difference between PAR(II) and PAR is that the former relates to the quantum absorption *rate* of PS II, which is independent of Chl content, whereas the latter represents a quantum *flux density* (or photon fluence rate), from which a PS II quantum absorption rate can be calculated only, if the PS II content is known.

Consequently, based on PAR(II), also a wavelength- and sample-dependent ETR(II) can be defined

4 $$ {\text{ETR}}({\text{II}}) = {\text{PAR}}({\text{II}}) \cdot \frac{{{\text{Y}}({\text{II}})}}{{{\text{Y}}({\text{II}})_{\max } }}, $$

where PAR(II) is the rate of quantum absorption at PS II, Y(II) the effective PS II quantum yield derived from the fluorescence ratio parameter ($F^{\prime}_{\text{m}}$ − *F*)/$F^{\prime}_{\text{m}}$, Y(II)_max_ the PS II quantum yield in the quasi-dark reference state under which Sigma(II)_λ_ was determined and ETR(II) the rate of electron transport expressed in units of electrons/(PS II s).

At very low light intensity, Y(II) approaches Y(II)_max_, so that Y(II)/Y(II)_max_ = 1 and ETR(II) = PAR(II). This means that in this state there is no loss of PS II efficiency with respect to the reference quasi-dark state (all centers open, non-energized, weak FR background illumination) under which Sigma(II)_λ_ was measured. Y(II)_max_ corresponds to the PS II quantum yield of a sample in the same state as given for measurement of *k*(II), which equals *F*_v_/*F*_m_. In measurements with algae and cyanobacteria, which display a relatively high level of PQ-reduction in the dark, it is advisable to measure *F*_v_/*F*_m_ in the presence of FR background light, which oxidizes the PQ-pool and induces the high PS II-efficiency state 1. FR background light is also routinely used for assessment of *k*(II) and Sigma(II)_λ_ via the *O*–*I*_1_ rise kinetics.

When compared with the common definition of rel.ETR in Eq. 2, it is apparent that the ETR-factor is contained in PAR(II) and that ETR(II) has the dimension of a turnover rate per PS II, whereas rel.ETR commonly has been treated as an electron flux density (or fluence rate), i.e., a rate per area, which without information on PS II per area must be considered hypothetical. In contrast, ETR(II) realistically describes the mean absolute rate of charge-separation per PS II in all PS II contained in the 1-mL illuminated sample.

**Fig. 8:**
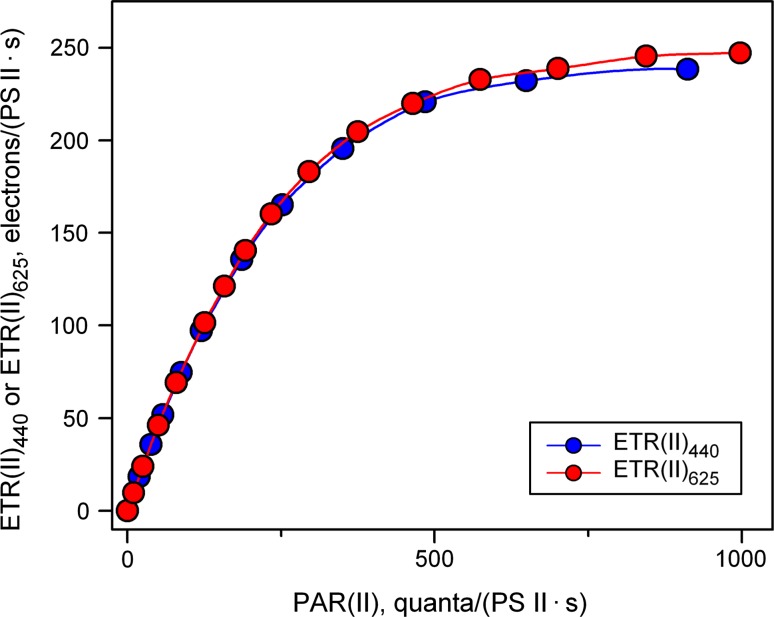
ETR(II) LC of a dilute suspension of *Chlorella* (300 μg Chl/L) using 440- and 625-nm light derived from the original LC of rel.ETR depicted in Fig. 4 by transformation of the PAR-scale into a PAR(II) scale using Eqs. 3 and 4 (see text). Illumination time at each intensity-setting was 3 min. Sigma(II) values of 4.547 and 1.669 nm^2^ were applied for 440 and 625 nm, respectively. In the calculation of ETR(II)_440_ and ETR(II)_625_, *F*_v_/*F*_m_ values of 0.68 and 0.66 were used, respectively. For comparison of the corresponding LC without PAR transformation, see Fig. 4

When the appropriate wavelength- and sample-dependent Sigma(II)_λ_ value is known, the user software of the multi-color-PAM supports the transformation of PAR into PAR(II). A practical example of transformation of a PAR-scale into a PAR(II) scale is given in Fig. 8, which is derived from the original rel.ETR LC data of Fig. 4 using the information on the values of Sigma(II)_λ_ measured with the same dilute *Chlorella* suspension briefly before the LC recording. PAR values were transformed into PAR(II) using Eq. 3 and ETR(II) was calculated according to Eq. 4.

In contrast to the rel.ETR LC of Fig. 4, where rel.ETR_max_ was much higher for 625 nm than for 440 nm, the ETR(II)_max_ values in Fig. 8 are almost identical for both the colors, thus confirming that the observed differences in rel.ETR are almost exclusively due to differences between Sigma(II)_440_ and Sigma(II)_625_. This may be considered strong support for the validity of Sigma(II)_λ_ determination via *O*–*I*_1_ measurements with the multi-color-PAM and its analysis by the *O*–*I*_1_ Fit approach. As the maximal value of ETR(II)_440_ is slightly lower than that of ETR(II)_625_, the question remains whether even after transformation of PAR into PAR(II), i.e., for identical rates of PS II turnover, blue light causes somewhat more photoinhibition (or down-regulation) than red light. For evaluation of these results it has to be considered that the illumination periods during the LC recording were relatively short (3 min), so that the time of exposure to potentially photoinhibitory intensities was relatively short. This aspect is further investigated in the following section.

When information on PS II concentration is available, it is possible to derive from ETR(II) a rough estimate of the absolute O_2_ evolution rate in units of mmol O_2_/(mg Chl s) using the following general equation:

5 $$ r{\text{O}}_{2} = \frac{{{\text{ETR}}({\text{II}})}}{{{\text{PSU}} \cdot ne ( {\text{O}}_{ 2} )\cdot M({\text{Chl}})}}, $$

where PSU is the photosynthetic unit size (i.e., number of Chl molecules per electron transport chain), *M*(Chl) is the molecular weight of Chl (approximately 900 g/mol) and *ne*(O_2_) the number of electrons required for evolution of 1 molecule of O_2_ (normally assumed to be 4). The absolute rate in the common units of μmol O_2_/(mg Chl h) is obtained by multiplication with 1,000 × 3,600. If PSU = 1,000 is assumed, the numerical value of the denominator amounts to 1,000 × 3,600, which means that in this case the numerical values of ETR(II) in electrons/(PS II s) and *r*O_2_ in μmol O_2_/(mg Chl h) are identical.

### Comparison of photoinhibition by 440- and 625-nm illumination

The *Chlorella* cells used in this study were cultured at relatively low ambient light intensities in the order of 20–30 μmol quanta/(m^2^ s) PAR, which may be compared with the *I*_*k*_ values of *Chlorella*, i.e., with the PAR values were light saturation sets in (see Fig. 5) that were 80 and 214 μmol/(m^2^ s) for 440 and 625 nm, respectively. The maximal intensities applied in the experiment of Figs. 4, 5, and 8 amounted to 1,000 μmol/(m^2^ s) for both the colors. Hence, in view of the up to about 50 times higher light intensities during LC recordings compared to growth conditions, photoinhibitory damage would not be surprising. In discussing Fig. 8, the question was raised, whether the slightly lower ETR(II)_max_ values with 440 nm compared to 625 nm could be due to a somewhat stronger photoinhibitory effect of 440 nm, as predicted by the two-step hypothesis of photoinhibition (see “Introduction”). This question can be further investigated by comparative measurements of dark–light–dark induction curves with repetitive assessment of effective PS II quantum yield, Y(II), where *Chlorella* is exposed for a longer period of time (22 min) to relatively high intensities of 440- and 625-nm light.

**Fig. 9:**
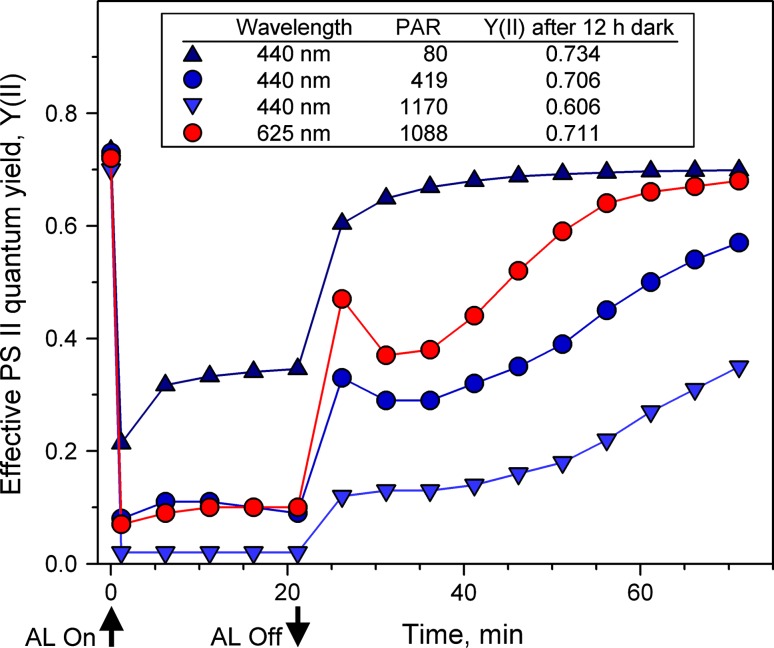
Changes of effective quantum yield, Y(II), induced during 22-min illumination with 440- and 625-nm light in dilute suspensions of *Chlorella* (300 μg Chl/L) followed by 50-min dark-regeneration. AL was switched on 40 s after measurement of *F*_v_/*F*_m_ (at time 0) and SP were applied every 5 min, starting 20 s after onset of AL. Use of the Script-file photoinhibition_Chl01.prg, with settings of light color and AL-intensity varied. PAR values are indicated in μmol quanta/(m^2^ s)

The data in Fig. 9 were obtained by automated measurements of slow kinetics under the control of a “Script-file” (see “Materials and methods”) programmed for initial measurement of *F*_v_/*F*_m_ = Y(II)_max_ and 22 min continuous illumination followed by 50-min dark-regeneration, with SPs applied every 5 min for determination of effective PS II quantum yield, Y(II). The 22-min continuous illumination served as photoinhibitory treatment and during the 50 min following this treatment the multi-phasic recovery of Y(II) was monitored. The Script was run four times with fresh samples using three different intensities of 440 nm and a single intensity of 625-nm light. The PAR of the 625-nm light was chosen such that it induced close to the same rate of PS II turnover as the medium intensity of the 440-nm light, i.e., the same PAR(II) was applied, as derived by Eq. 3 (in the given example, 419 × 4.547 almost equals 1,088 × 1.669).

Comparison of the three curves with 440-nm illumination (dark-blue curve at top and two light-blue curves at bottom of Fig. 9) provides some insight into light-induced suppression of Y(II) in *Chlorella*. At 80-μmol/(m^2^ s) (top curve, corresponding to *I*_*k*_, i.e., near the beginning of saturation) after its initial suppression Y(II) gradually increases during illumination, reflecting light-activation of the Calvin–Benson cycle. Upon darkening, Y(II) returns with biphasic kinetics within 50 min to its original dark-level. In contrast, at 419 μmol/(m^2^ s) (third curve from top) not only the initial suppression of Y(II) is more pronounced but also after about 10 min there is a gradual decline of Y(II), which suggests that light-activation of the Calvin–Benson cycle cannot prevent gradually increasing inhibition of PS II. Upon darkening, a rapid phase of Y(II) recovery is followed by a dip phase, before Y(II) slowly returns toward its dark-level, with distinctly slower kinetics than in the experiment using 80 μmol/(m^2 ^s) and 80 % dark-recovery within 50 min. Finally, at 1,022 μmol/(m^2 ^s) of 440-nm light (bottom curve), Y(II) is suppressed to values close to zero during illumination and the recovery upon darkening displays two phases separated by a wide plateau, with 50-min dark-recovery amounting to 49 % only. After 12-h dark-recovery, the Y(II) of all samples except for the one illuminated at 1,170 μmol/(m^2 ^s) recovered to values close to the original *F*_v_/*F*_m_ (see inset of Fig. 9).

The red curve in Fig. 9 shows the responses observed when almost identical PAR(II) of 625-nm light is applied (1,088 μmol/(m^2 ^s)) as in the measurement with the intermediate 440-nm intensity (419 μmol/(m^2 ^s)). Hence, at equal PAR(II), the responses of 440- and 625-nm quanta are very similar, even when applied over a longer period of time. At the end of illumination the Y(II) with 625 nm is just marginally higher than with 440 nm, similarly as in the LC-recordings of Fig. 8. There are, however, remarkable differences in the dark-recovery kinetics. After 625-nm illumination, the dark-recovery of Y(II) is distinctly faster than after 440-nm illumination, amounting to 97 % after 50 min. This shows clearly that the PS II turnover is not the only parameter affecting the quantum yield of PS II. Obviously, 440 nm can lower the PS II quantum yield by an additional mechanism, which is induced at high light intensity and still is effective after 50-min dark-recovery.

## Concluding discussion and outlook

The presented data demonstrate that the new multi-color-PAM is more than just another PAM fluorometer offering various colors of light. The new dimension of this device relates to the precision, flexibility, and speed, with which the various colors of light can be applied, with the main aim of obtaining quantitative information on the rate of wavelength-dependent charge-separation in PS II reaction centers. This aim was reached via new developments at the levels of opto-electronics, microprocessor-based firmware and user software. Recent technical progress in LED technology made it possible to develop an extremely powerful miniature light source, which provides all the essential light qualities, for which in former days a whole bench of high-power light sources and flash-discharge lamps (or lasers) would have been required. With six separate colors of ML, six colors of AL (also serving for ST and MT flashes) and FR light, a total of 13 independent light sources are integrated on the 10 × 10 mm area of the multi-color-COB array. Such compact design enables optimal coupling of the light source to the 10 × 10 mm sample cuvette, assuring identical optical pathways of the various types of light. Close to optimal optical conditions are possible by use of low cell densities, as excellent signal/noise ratios are obtained at 200–300 μg Chl/L, where light-intensity gradients are negligibly small. Operation of this complex measuring system is facilitated by automated measuring routines, which assure reproducibility and prevent operator’s errors.

The novel information provided by the new device is contained in the wavelength-dependent parameter Sigma(II)_λ_, the definition of which for technical–methodological reasons differs from the parameter σ_PSII_ used by researchers in limnology and oceanography (Koblizek et al. 2001; Kolber et al. 1998). Almost all σ_PSII_ values reported in the literature were determined for one color of light, irrespective of the pigment-composition of the investigated sample. Furthermore, σ_PSII_ has been measured in widely differing states of the sample, with the PS II acceptor side being more or less reduced, which leads to corresponding changes in the sigmoidicity and time constant of the light-induced fluorescence rise. In contrast, Sigma(II)_λ_ is always measured in a defined quasi-dark reference state, at close to maximal efficiency of PS II. Any changes of the sample with respect to this reference state, e.g., by light-driven down-regulation or photodamage of PS II, do not affect Sigma(II)_λ_, but are contained in the effective PS II quantum yield, Y(II), which is lowered with respect to the PS II quantum yield, Y(II)_max_, measured in the reference state, in which also Sigma(II)_λ_ was measured. Therefore, the values of Sigma(II)_λ_ obtained for *Chlorella* and *Synechocystis* are substantially higher than the σ_PSII_ values reported, e.g., by Koblizek et al. (2001).

Other new parameters introduced for work with the multi-color-PAM are PAR(II) and ETR(II), which describe the *absolute* rates of photon absorption by PS II and electron transport via PS II, respectively. PAR(II) just like Sigma(II)_λ_ is defined for a quasi-dark reference state. With this approach, fluorescence-based estimation of absolute photosynthetic electron transport rates in optically thin suspensions has been given a reliable methodological basis. Related work using the parameter σ_PSII_ can be found almost exclusively in the limnology and oceanography literature, which partially may be due to the complexity of its definition, understanding of which requires considerable background knowledge. Comparison of Figs. 4 and 8 demonstrates convincingly that quantitative information on the functional PS II absorption cross section is of general importance for quantitative assessment of photosynthetic activity, which becomes very evident as soon as different colors of light are applied.

It may be foreseen that the multi-color-PAM will stimulate future research of the wavelength dependence of photosynthesis not only in suspensions of algae and cyanobacteria but also in whole leaves, macrophytes or even corals and other organisms containing endosymbionts. The example presented of apparent differences in photoinhibition by close to identical PAR(II) of 440- and 625-nm light in *Chlorella* demonstrates the methodological value of Sigma(II)_λ_ determination with this new device in a range of basic and applied plant physiological applications. Adjustment of close to equal PAR(II) should be also possible with leaves and other optically dense samples. When fluorescence is excited by 440-nm ML and *F* < 710 nm is measured, almost selectively fluorescence responses of the uppermost cell layers are measured (Schreiber et al. 2011), so that differences due to varying depths of penetration can be avoided. This is an example for the advantage of optional use of separate colors for measuring and actinic light. Rappaport et al. (2007) pointed out the advantages of using green light (both measuring and actinic) to minimize light-intensity gradients. However, even with green light substantial gradients persist and, most importantly, the photosynthetic performance of different cell layers within a leaf (as well as other types of optically dense samples) is heterogeneous and their responses should not be mixed up. Therefore, to assess, e.g., differences between adaxial and abaxial leaf sides it is better to employ *strongly absorbed* ML (e.g., 440 nm), so that the response is restricted to the uppermost layers of cells, which may be considered close to homogenous (Schreiber et al. 2011).

The data of Fig. 9 were presented as one example of practical application of the new multi-color device to induce defined rates of quanta absorption in PS II using different colors. These measurements may be considered particularly reliable, as they were carried out with dilute suspensions, i.e., with negligibly small PAR-gradients. The data demonstrate distinct differences between post-illumination responses after close to identical absorption of 440- and 625-nm quanta, the direction of which in principle does agree with the two-step hypothesis of photoinhibition. Specific absorption of blue light could cause damage of the Mn-cluster of the OEC, resulting in donor-side limitation of PS II, production of ROS and secondary damage of various enzymatic reactions, including repair of PS II reaction centers (Ohnishi et al. 2005; Hakala et al. 2005; Nishiyama et al. 2006). However, this may not be the only mechanism that can explain the observed differences between 440- and 625-nm light. More extensive measurements, using longer illumination times and inhibition of the simultaneously occurring repair reactions, will be required for conclusive evidence. In any case, it is clear that the multi-color-PAM does offer the potential for quantitative investigation of the wavelength dependence of photoinhibition, particularly when combined with other promising new measuring techniques (Chow et al. 2005; Matsubara and Chow 2004).

Besides the mechanism of photodamage to PS II, other important topics relating to wavelength-dependent effects on the photosynthetic apparatus are reversible state 1–state 2 transitions (Mullineaux and Emlyn-Jones 2005) and NPQ induced in cyanobacteria via blue-light absorption by the orange carotenoid protein (Kirilovsky 2007). The multi-color-PAM appears well suited for detailed investigations of these fascinating adaptational processes (Bernát et al. 2012).

## Abbreviations

AL: Actinic light
COB: Chip on board
ETR: Electron transport rate
ETR(II): Absolute rate of PS II turnover, electrons/(PS II s)
*F*_m_: Dark-acclimated maximal fluorescence yield
$F^{\prime}_{\text{m}}$: Maximal fluorescence yield during illumination
*F*_o_: Dark-acclimated minimal fluorescence yield
FR: Far-red light (>700 nm)
FRR: Fast repetition rate
*F*_v_: Variable fluorescence yield
*I*_*k*_: Light intensity at onset of saturation (μmol quanta/(m^2^ s))
*J*: Sigmoidicity/connectivity parameter
*k*(II): Rate constant of PS II turnover (1/ms)
LC: Light curve of fluorescence parameters
LED: Light-emitting diode
MF: Frequency of pulse-modulation
ML: Pulse-modulated measuring light
MT: Multiple-turnover light pulse
NPQ: Non-photochemical quenching
OEC: Oxygen-evolving complex
*O–I*_1_: Photochemical phase of fast fluorescence rise
PAM: Pulse amplitude modulation
PAR: Photosynthetically active radiation (μmol quanta/(m^2^ s))
PAR(II): Absolute rate of PS II turnover, quanta/(PS II s)
PPFR: Photosynthetic photon fluence rate (μmol quanta/(m^2^ s))
Sigma(II)_λ_: Wavelength-dependent cross section of PS II (nm^2^)
SP: Saturation pulse
ST: Single-turnover light pulse
Tau: Time constant of light-driven Q_A_-reduction (ms)
Tau(reox): Time constant of Q_A_ reoxidation (ms)
Y(II): Effective quantum yield of PS II
σ_PSII_: Functional absorption cross section of PS II (nm^2^)
